# Porokeratosis ptychotropica*

**DOI:** 10.1590/abd1806-4841.20164399

**Published:** 2016

**Authors:** Ana Carolina Franco Tebet, Tatiana Gandolfi de Oliveira, Anna Rita Ferrante Mitidieri de Oliveira, Fabiolla Sih Moriya, Jayme de Oliveira Filho, Luiz Carlos Cucé

**Affiliations:** 1 Universidade de Santo Amaro (UNISA) – Santo Amaro (SP), Brazil

**Keywords:** Dermatology, Dermopathology, Pathology, Porokeratosis

## Abstract

Porokeratosis is a skin disorder clinically characterized by annular plaques with keratotic borders resembling the Great Wall of China and histopathologically by cornoid lamellae. The disease has several clinical variants. Porokeratosis ptychotropica, which has recently become part of these variants, is quite rare and little known. The entity is characterized by verrucous plaques – which may resemble a psoriasis plaque – that affect the regions of the buttocks, most commonly the gluteal cleft, with or without extremity involvement. Itching is often present. We report a rare case of porokeratosis ptychotropica and highlight its unusual manifestation (single plaque), the first case reported in the Brazilian literature.

## INTRODUCTION

Porokeratosis is a skin disorder clinically characterized by annular plaques with keratotic border resembling the Great Wall of China and and histopathologically by cornoid lamellae.^1-8^ The disease may assume different clinical forms – which are mostly well known – and its variants are classified as plaque-type or porokeratosis of Mibelli, palmoplantar, linear, and punctate porokeratosis.^1,2,3,6,7,8^

However, a quite rare and little known subtype of the disease has recently become part of these variants: porokeratosis ptychotropica.^5^ The entity is characterized by verrucous plaques – which may resemble a psoriasis plaque – that affect the regions of the buttocks, most commonly the gluteal cleft, with or without extremity involvement. Itching is often present. Due to its morphology, it was also named hyperkeratotic porokeratosis, genitogluteal porokeratosis, porokeratoma, and follicular porokeratosis.^1,4,8^

Risk factors for the disease include exposure to ultraviolet radiation, organ transplantation, chemotherapy, repetitive trauma, liver failure, chronic renal failure, hepatitis C, HIV, and other diseases associated with immunosuppression. However, its etiology is not well established.^9^

## CASE REPORT

**Figure 1:**
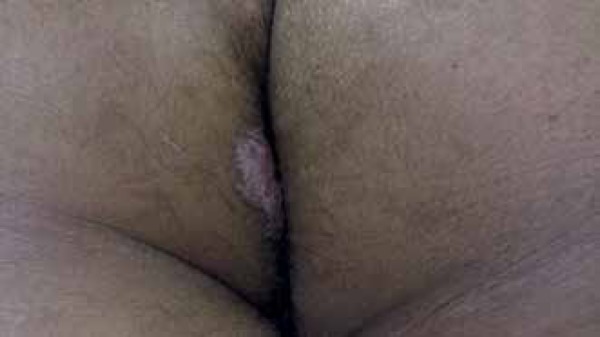
Patient presented with oval erythematous plaque with vegetating surface, slightly hypochromic and with well-defined rete ridge-like edges in the gluteal region

**Figure 2:**
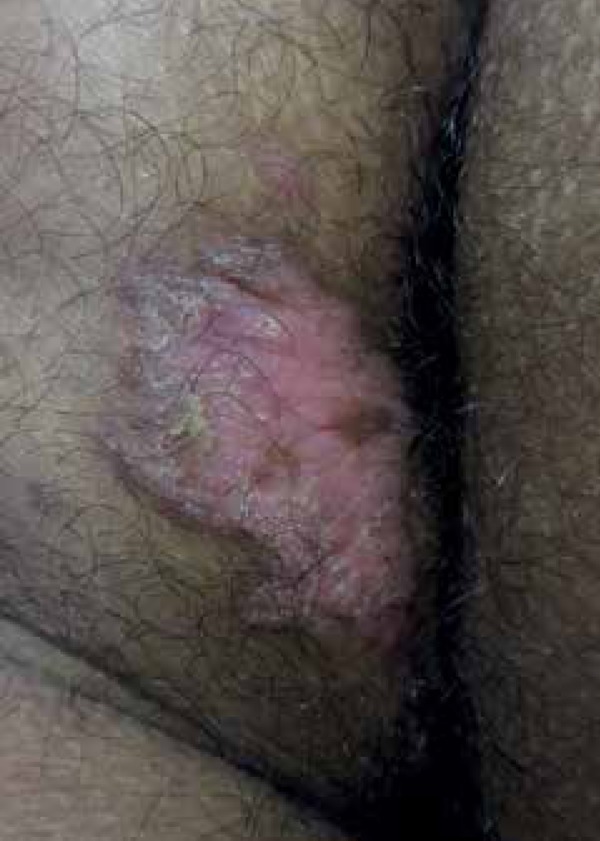
Greater detail of the oval erythematous plaque with vegetating surface, slightly hypochromic and with well- defined rete ridge-like edges

We report a 23-year-old male mulatto patient complaining of pruritus and lesions in the right gluteal region for 9 years. The patient observed no lesions at the beginning, only itching. However, he developed an erythematous plaque of slow growth, which now appears as an oval verrucous plaque, slightly hypochromic, with well-defined thread-like borders of approximately 7cm x 4cm in diameter (Figures 1 and 2). The patient reported no involution of the lesion during that period. Skin examination revealed no other changes. The patient had no history of systemic, liver, or kidney diseases. He denies exacerbated sun exposure or any immunodeficiency. Family history revealed no similar lesions. He reported having received topical treatment with corticosteroids with no improvement based on a misdiagnosed psoriasis.

Diagnostic hypotheses included cutaneous tuberculosis, cromomycosis, paracoccidioidomycosis, leishmaniasis, squamous cell carcinoma, condyloma, and neurodermatitis.

**Figure 3:**
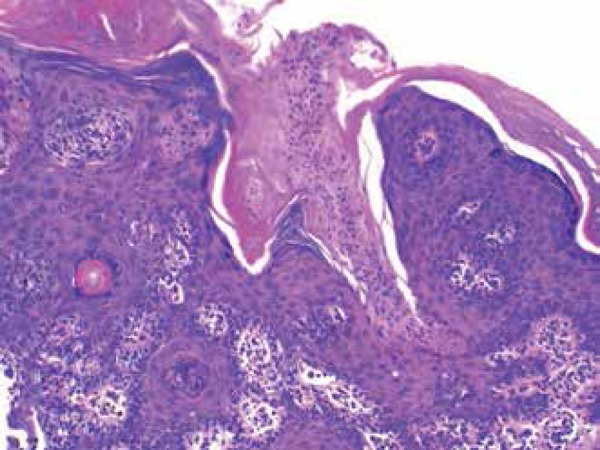
Histopathology (HE staining 40x) revealed regular thickening of the skin with mild papillomatosis and agranulosis sections topped by broad parakeratotic columns, featuring cornoid lamellae

A punch biopsy revealed regular thickening of the skin with mild papillomatosis and agranulosis sections topped by broad parakeratotic columns, featuring cornoid lamellae (Figure 3).

We started treatment with antihistamine – considering the remarkable itching – until we received the results of the anatomapathologic examination. After the diagnosis of porokeratosis ptychotropica, we started treatment with topical tretinoin 0.5%. The disease remains unresponsive to treatment due to the short time (1 week).

## DISCUSSION

Porokeratosis ptychotropica is a quite rare and little known disease with difficult diagnosis.^1^ It was first reported in 1985 by Helfman and Poulos, who described it as reticular porokeratosis affecting the genital/pelvic region. Ten years later, Lucker *et al.* named the disease as “porokeratosis ptychotropica”, a porokeratotic lesion involving the gluteal region associated with severe itching presenting a different (verrucous) morphology.^1,3^ A few cases have been reported since then, sometimes involving both sides of the buttock.^2,5^

Out of the 22 cases reviewed by Takiguchi *et al.* in 2010, 90% were male with a mean age of 46.7 years (between 27-84 years). The main affected regions were: the buttocks (36.36%); genitogluteal region (31.82%); and buttocks region with extremity involvement (22.7%).2 However, porokeratosis ptychotropica is more commonly described as numerous coalescing plaques accompanied by satellite lesions, unlike our case that presented a solitary plaque with no satellite lesions.^5,9^

Therapy with good response is rather poor. Tentative therapies include 5-fluorouracil, PUVA, Imiquimod and even CO2 laser.^1,2,3,8^ The only case of successful therapy described in the literature was with the dermatome after lesion whitening with CO2 laser treatment, but with subsequent relapse. In that same study, the use of 5-fluorouracil only decreased the lesion and relieved pruritus.^1,3,4,8^ However, steroids, PUVA, calcipotriol, tacrolimus, imiquimod, vitamin A and cryotherapy failed treatment.^1,2,3,8,9^

Our patient has been using tretinoin 0.5% for one week, but with no improvement so far. Since it is a single lesion in the gluteal cleft region, we chose not to use imiquinod or 5-fluorouracil, which could injure the contralateral healthy skin.

It is worth mentioning that the malignant transformation of the porokeratotic lesion can occur in 7.5% of cases, mostly associated with long-term, linear, large-size lesions.^1,2,3,4,8^

The importance of our work is related to the rarity of the disease, as well as to its unusual manifestation (single plaque). It is the first case reported in the Brazilian literature. We hope this report can help elucidate undiagnosed cases so that physicians opt for the ideal therapy, which will provide comfort to the patients and prevent malignant transformations.

We suggest a unification of the nomenclature, as well as the inclusion of porokeratosis ptychotropica as a subtype of porokeratosis since it is still not considered as such by some authors. Therefore, it will be more easily remembered in cases of differential diagnosis of perianal rash.
